# Mass Spectrometry-Based Proteome Profiling of Extracellular Vesicles Derived from the Cerebrospinal Fluid of Adult Rhesus Monkeys Exposed to Cocaine throughout Gestation

**DOI:** 10.3390/biom12040510

**Published:** 2022-03-28

**Authors:** Hilal A. Rather, Shalini Mishra, Yixin Su, Ashish Kumar, Sangeeta Singh, Biswapriya B. Misra, Jingyun Lee, Cristina M. Furdui, Lindsey R. Hamilton, Robert W. Gould, Susan H. Nader, Michael A. Nader, Gagan Deep

**Affiliations:** 1 Department of Cancer Biology, Wake Forest School of Medicine, Winston-Salem, NC 27157, USA; hrather@wakehealth.edu (H.A.R.); samishra@wakehealth.edu (S.M.); ysu@wakehealth.edu (Y.S.); ashish.kumar@wakehealth.edu (A.K.); sasingh@wakehealth.edu (S.S.); 2 Independent Researcher, Pine-211, Raintree Park Dwaraka Krishna, Namburu 522508, Andhra Pradesh, India; bbmisraccb@gmail.com; 3 Wake Forest Baptist Comprehensive Cancer Center, Wake Forest School of Medicine, Winston-Salem, NC 27157, USA; jilee@wakehealth.edu (J.L.); cfurdui@wakehealth.edu (C.M.F.); 4 Department of Internal Medicine, Section on Molecular Medicine; Wake Forest School of Medicine, Winston-Salem, NC 27157, USA; 5 Department of Physiology and Pharmacology, Wake Forest School of Medicine, Winston-Salem, NC 27157, USA; lindsey.hamilton@ucdenver.edu (L.R.H.); rgould@wakehealth.edu (R.W.G.); snader@wakehealth.edu (S.H.N.); 6 Center for Addiction Research, Wake Forest School of Medicine, Winston-Salem, NC 27157, USA

**Keywords:** cocaine, extracellular vesicles, cerebrospinal fluid, biomarker, mass spectrometry

## Abstract

Cocaine use disorder has been reported to cause transgenerational effects. However, due to the lack of standardized biomarkers, the effects of cocaine use during pregnancy on postnatal development and long-term neurobiological and behavioral outcomes have not been investigated thoroughly. Therefore, in this study, we examined extracellular vesicles (EVs) in adult (~12 years old) female and male rhesus monkeys prenatally exposed to cocaine (*n* = 11) and controls (*n* = 9). EVs were isolated from the cerebrospinal fluid (CSF) and characterized for the surface expression of specific tetraspanins, concentration (particles/mL), size distribution, and cargo proteins by mass spectrometry (MS). Transmission electron microscopy following immunogold labeling for tetraspanins (CD63, CD9, and CD81) confirmed the successful isolation of EVs. Nanoparticle tracking analyses showed that the majority of the particles were <200 nm in size, suggesting an enrichment for small EVs (sEV). Interestingly, the prenatally cocaine-exposed group showed ~54% less EV concentration in CSF compared to the control group. For each group, MS analyses identified a number of proteins loaded in CSF-EVs, many of which are commonly listed in the ExoCarta database. Ingenuity pathway analysis (IPA) demonstrated the association of cargo EV proteins with canonical pathways, diseases and disorders, upstream regulators, and top enriched network. Lastly, significantly altered proteins between groups were similarly characterized by IPA, suggesting that prenatal cocaine exposure could be potentially associated with long-term neuroinflammation and risk for neurodegenerative diseases. Overall, these results indicate that CSF-EVs could potentially serve as biomarkers to assess the transgenerational adverse effects due to prenatal cocaine exposure.

## 1. Introduction

Currently, substance use disorder (SUD) is one of the serious health concerns in the United States. According to National Survey on Drug Use and Health Report 2020, 20.4 million people aged 12 years or older were reported to have SUD in 2019. Among them, 5.5 million people were cocaine users. The number of people aged 12 or older in 2019 who initiated cocaine use averaged >1800 people each day, with 671,000 people for the entire year [1]. The rate of deaths from drug overdose involving cocaine has increased in recent years in the United States. According to the data from the National Vital Statistics System, the rate of drug overdose deaths involving cocaine has nearly tripled from 1.6 per 100,000 in 2013 to 4.5 in 2018 [2].

The use of cocaine by females during pregnancy is reported to have long-term transgenerational effects on their offspring [3]. It is estimated that more than 5% of pregnant women in the United States use one or more addictive substances [4]. Notably, most women who have cocaine-use disorders (CUDs) are of childbearing age. There are around 750,000 cocaine-exposed pregnancies every year (Center for Behavioral Health Statistics and Quality, 2015). The magnitude of the effects of prenatal cocaine exposure will depend upon dosage amount and duration of exposure. Cocaine use during pregnancy is associated with several physical deficits to the newborn, including reduced body weight, brain weight, body length, and head circumference at birth [3,5]. Cocaine readily crosses the placenta, thereby affecting neuronal maturation and increasing the risk for central nervous system disruptions during fetal development [6,7]. In addition, it is believed to produce cognitive deficits, physiological and behavioral abnormalities. Anxiety and depression are the most commonly observed phenotype in the subsequent generation following cocaine exposure in parents [8].

Hamilton et al. [9,10] have characterized the behavioral and neuropharmacological impact of prenatal cocaine exposure in adult male and female rhesus monkeys. These investigators reported that prenatally cocaine-exposed monkeys required a significantly greater number of sessions to reach criterion performance in an operant food-reinforced paradigm compared with control monkeys. However, no differences were observed in the concentrations of the monoamine metabolites homovanillic acid and 5-hydroxyindole acetic acid in CSF samples [9]. Further, dopamine receptor functions were studied in these same cocaine-exposed monkeys and controls. The results suggested that there were no significant changes in the dopamine D1 receptor agonist-elicited behaviors and in D2-like receptor availability, using PET imaging. However, there was evidence that prenatal cocaine exposure resulted in long-term effects on dopamine D3 receptor function in adults [10]. Compared to the large number of studies involving current cocaine users, the effects of cocaine use during pregnancy on postnatal development and long-term neurobiological and behavioral outcomes have been less thoroughly investigated. In that respect, a particular advantage of the monkey studies described above is the standardization of in utero cocaine exposure for each monkey, which lasted for the duration of gestation (~26 weeks) [11]. Another major challenge is the lack of standardized biomarkers, which could be used for early identification and monitoring of deficits and abnormalities in individuals exposed to cocaine during gestation.

Recent studies have shown that various molecular biomarkers could be useful in predicting the susceptibility to CUD or in assessing the adverse effects of cocaine exposure [12,13,14]. For example, overexpression of miR-212 in the dorsal striatum of rats resulted in an attenuation of cocaine self-administration [13]. Similarly, the expression of miR-431 was significantly elevated in the dorsal striatum region of cocaine-susceptible rats compared to cocaine-resilient rats [14]. Lately, extracellular vesicles (EVs) have emerged as an attractive nano-tool for identifying molecular biomarkers associated with drug abuse and associated side effects [12,15,16,17]. For example, Shahjin et al. identified distinct brain-derived EVs miRNA signatures associated with in utero and postnatal oxycodone exposure [15]. Li et al. identified 10 key miRNAs in serum EVs associated with the development of addiction to methamphetamine and ketamine in rats [16]. We have recently reported that brain-derived exosomes from the plasma of monkeys self-administering oxycodone provided evidence to predict the pro-inflammatory and neurodegenerative consequences of oxycodone exposure [17]. Hence, EVs could be used to identify biomarkers associated with adverse effects caused by cocaine exposure during pregnancy; however, to the best of our knowledge, no such study has yet been performed. Therefore, in the present study, we characterized EVs isolated from the CSF of adult rhesus monkeys who were exposed to cocaine throughout gestation and control monkeys. Importantly, these adult monkeys had a minimal drug history, other than the in utero cocaine exposure prior to the start of this study. We hypothesize that these EVs could be used as potential molecular biomarkers for the detection of adverse effects in individuals exposed to cocaine during pregnancy.

## 2. Materials and Methods

*Subjects:* The subjects were 20 adult rhesus monkeys (*Macaca mulatta*), born between 1993 and 1995 and raised at the FDA facility in Little Rock, AR, until 2007 when they were transferred to Wake Forest School of Medicine. Of the 20 monkeys, 11 (7 males and 4 females) were prenatally exposed to cocaine and 9 were controls (4 males and 5 females); the control monkeys were exposed to saline throughout gestation, as described previously [18,19]. Briefly, the mothers of the monkeys used in this study received intramuscular injections of escalating doses of cocaine or saline. For the cocaine group, prior to mating, mothers received a dose of 1.0 mg/kg/injection cocaine, which was increased throughout gestation to up to 8.5 mg/kg/injection; these injections were given three times per day for the entire course of gestation, with mean cumulative cocaine intake of 1092.3 (±59.8 SEM) mg/kg [18]. Other than their prenatal drug histories, all monkeys had nearly identical experimental histories (see [11,18]). Monkeys were individually housed in stainless-steel cages with water available ad libitum and had visual and auditory contact with each other. All experimental and environmental enrichment protocols were approved by the Wake Forest University Institutional Animal Care and Use Committee. All manipulations were performed in accordance with the 2011 National Research Council Guidelines for the Care and Use of Mammals in Neuroscience and Behavioral Research.

*Collection of CSF*: For CSF collection, monkeys were anesthetized with 10 mg/kg ketamine, the neck and the back of the skull were shaved and cleaned with betadine and 95% ethanol. A 25-gauge, 1.5-in. needle attached to a 3-mL syringe was inserted through the cisterna magna, and approximately 2 mL of CSF was removed within 10 min of induction of anesthesia. All samples were collected in the morning, at approximately the same time of day (0900 h). The samples were immediately transferred to vacutainer tubes on ice. Samples were centrifuged at 4 °C for 30 min at 3000 rpm and then aliquoted into microcentrifuge tubes for storage at −30 °C until further use.

*EVs isolation from CSF*: EVs were isolated from CSF using Exoquick-TC (System Biosciences, Palo Alto, CA, USA) following the vendor’s instruction. Briefly, CSF was centrifuged at 3000× *g* for 15 min to remove cells and cell debris. Next, the supernatant was transferred to a sterile tube, and an appropriate volume of ExoQuick-TC was added and incubated overnight at 4 °C. Thereafter, tubes were centrifuged at 1500× *g* for 30 min, and the supernatant was removed. Tubes were centrifuged again at 1500× *g* for 5 min to remove any remaining supernatant or ExoQuick-TC solution. EV pellet was suspended in sterile 1X DPBS, and protein concentration was measured by NanoDrop.

*Nanoparticle tracking analyses* (*NTA*): Quantification of the hydrodynamic diameter distribution and concentration of EVs were performed using the Nanosight NS300 (Malvern Panalytical, Malvern, UK) equipped with a violet laser (405 nm) and running software version NTA3.2. The instrument was primed using phosphate-buffered saline (PBS), pH 7.4, and the temperature was maintained at 25 °C. Five measurements (30 s each) were obtained for each sample, and their average was plotted as representation of size distribution and concentration (particles/mL).

*Immunogold labeling and transmission electron microscopy* (*TEM*): For immunogold labeling, EVs were fixed with 2% paraformaldehyde in PBS buffer (pH 7.4), then adsorbed for 1 h to a carbon-coated grid. Samples were incubated with specific primary antibody; CD63 (antibodies-online Inc., Limerick, PA, USA) or CD9 or CD81 (Abcam, Waltham, MA, USA); followed by an appropriate secondary antibody tagged with gold particles (Sigma-Aldrich, St. Louis, MD, USA). EVs were stained with 1% uranyl acetate for 5 min. Images were captured on Tecnai T12 transmission electron microscope.

*Proteomics analyses by mass spectrometry* (*MS*): LC-MS grade Water (Cat. Num. W6-4), acetonitrile (Cat. Num. A955-4), LC-MS grade formic acid (Cat. Num. A117-50), and MS grade trypsin protease (Cat. Num. 90059) were purchased from Thermo Fisher Scientific (Waltham, MA, USA). A total of 12 samples, including 6 from the control and 6 from the prenatally cocaine-exposed monkeys (with *n* = 3/sex) were utilized for proteomic analyses. EVs suspended in PBS were lysed by adding an equal volume of 2X radioimmunoprecipitation (RIPA) buffer containing protease inhibitor cocktail. The protein extract was chemically reduced with 10 mM dithiothreitol and alkylated with 30 mM iodoacetamide followed by protein precipitation with cold acetone. The protein pellet was then suspended in 50 mM ammonium bicarbonate and proteolytically digested with trypsin at 37 °C overnight. Peptides were purified using a C18 spin column and prepared in 5% (*v*/*v*) acetonitrile in water containing 1% (*v*/*v*) formic acid for LC-MS/MS analyses. The analysis was performed on an Orbitrap Velos Pro Mass Spectrometer (Thermo Scientific, Waltham, MA, USA) coupled with a Dionex Ultimate-3000 nano-UPLC system (Thermo Scientific, Waltham, MA, USA) equipped with an Acclaim PepMap 100 (C18, 5 μm, 100 Å, 100 μm × 2 cm) trap column and an Acclaim PepMap RSLC (C18, 2 μm, 100 Å, 75 μm × 50 cm) analytical column. MS spectra were acquired by the top 10 data-dependent scans with a dynamic exclusion option of 30 s enabled.

Spectra were searched using Sequest HT within the Proteome Discoverer v2.2 (Thermo Scientific, Waltham, MA, USA) and UniProt human protein FASTA database (20,258 annotated entries, February 2018). The search parameters were as follows: FT-trap instrument, parent mass error tolerance of 10 ppm, fragment mass error tolerance of 0.6 Da (monoisotopic), variable modifications of 16 Da (oxidation) on methionine, and fixed modification of 57 Da (carbamidomethylation) on cysteine. Signal-to-noise ratio (STN) was determined following similar approaches, as published previously [20]. Proteins with STN values ≥0.3 and ≤−0.3 STN were selected for further analyses. Proteomics data was analyzed using Ingenuity Pathway Analysis (IPA) (QIAGEN Inc., https://www.qiagenbioinformatics.com/products/ingenuitypathway-analysis; accessed on 18 February 2022) as described earlier [21].

*Statistical analyses*: Statistics used for IPA (Ingenuity Pathway Analysis) can be found at the website http://www.ingenuity.com/index.html; accessed on 18 February 2022. Unsupervised principal component analysis (PCA) was performed using ggplot2 package.

## 3. Results

### 3.1. Characterization of CSF-EVs

**Figure 1:**
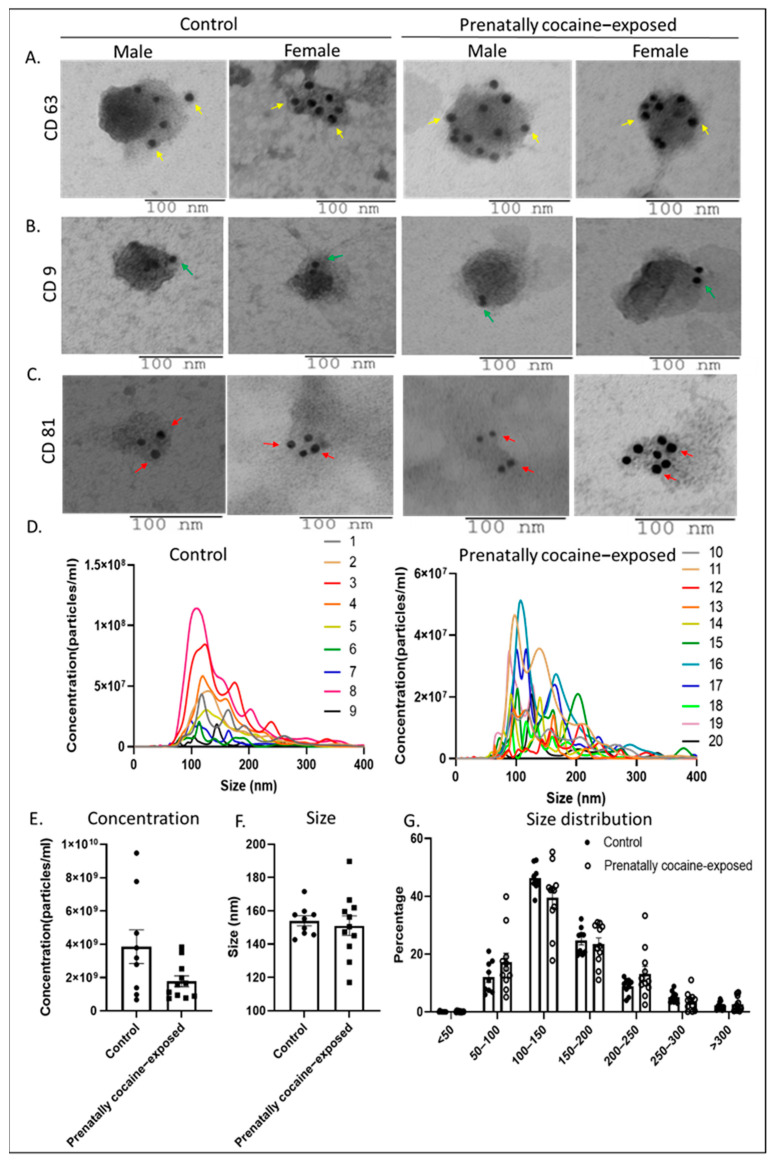
Characterization of CSF-EVs. (**A**–**C**) Representative immunogold labeling (using TEM) images of CSF-derived EVs from control and prenatally cocaine-exposed male and female monkeys are shown. The scale bar is shown at the bottom of each image. Yellow arrows indicate the gold particles bound to CD63 (**A**), green arrows indicate gold particles bound to CD9 (**B**) and red arrows indicate gold particles bound to CD81 (**C**) on the surface of CSF-EVs. (**D**) CSF-EVs from all the samples were analyzed for concentration (particles/mL) and size distribution by NTA and presented for control (*n* = 9) and prenatally cocaine-exposed monkeys (*n* = 11). Each line with a unique color represents the concentration and size distribution for an individual sample. NTA data was also analyzed for total particle concentration (number/mL) (**E**) and mean size (**F**) and presented in the bar graph. (**G**) Percentage size distribution is also presented for the control and prenatally cocaine exposed group. Data are presented as mean ± SEM for control (*n* = 9) and prenatally cocaine-exposed (*n* = 11) group.

We first characterized the isolated EVs by immunogold labeling and TEM for the surface expression of various tetraspanins (CD63, CD9, and CD81), which are the established biomarkers for small EVs (sEV). Results demonstrated that CD63, CD81, and CD9 (abundant on EV surface in the same order) were readily detected on the surface of EVs isolated from the CSF of male and female monkeys in both control and prenatally cocaine-exposed groups (Figure 1A–C). The TEM images also showed that the size of these EVs was in the range that is mostly assigned to sEV. Next, NTA was performed to assess the concentration (particles/mL) and size distribution of EVs. Figure 1D shows the concentration and size distribution of EVs from all control (left) and prenatally cocaine-exposed (right) monkeys, demonstrating a significant variation for this measure in each group. A prominent decrease (~54%) in total EV concentration (particle number/mL) was observed in the prenatally cocaine-exposed group compared to the control group; however, this difference did not achieve statistical significance (Figure 1E). NTA results also showed that the mean particle size of EVs was below 150 nm in both groups without any statistically significant difference (Figure 1F). Further analyses of NTA data based upon size distribution percentage (Figure 1G) showed that the majority of the particles were <200 nm in size (~83% in control and ~80% in the prenatally cocaine-exposed group), again highlighting enrichment of sEV in our preparation. We did not observe any sex-based differences in EVs characterization, so male and female data were combined for each group.

### 3.2. Proteomic Characterization of CSF-EVs by Mass Spectrometry (MS)

**Figure 2:**
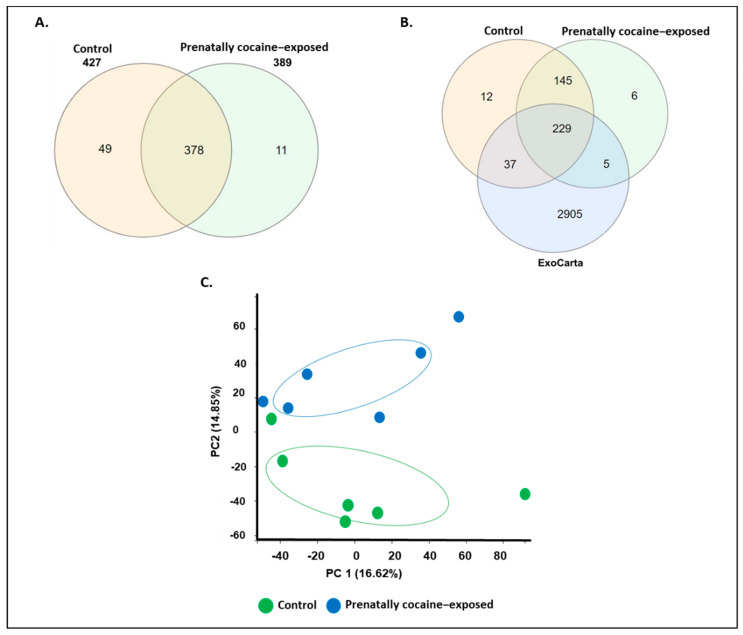
Characteristics of proteins loaded in CSF-EVs. (**A**) Venn diagram representing the proteins identified from control and prenatally cocaine-exposed groups. (**B**) Venn diagram representing the overlap of proteins identified in CSF-EVs in control and prenatally cocaine-exposed monkeys with ExoCarta dataset. (**C**) Principal component analysis (PCA) showing cocaine exposure-based separation of proteins.

Next, we characterized the cargo proteins loaded in CSF-EVs from control and prenatally cocaine-exposed monkeys by MS analyses. The number of proteins common or uniquely present in CSF-EVs from control and prenatally cocaine-exposed groups is shown in Figure 2A. A total of 427 proteins were found in control animals, whereas in prenatally cocaine-exposed monkeys, this number was 389. Furthermore, 378 proteins were common between groups, while 49 proteins were uniquely present in control monkeys and 11 proteins were unique to prenatally cocaine-exposed monkeys. Overall, the majority of proteins were present in CSF-EVs from both groups.

We further compared the identified EV proteins from each group to the existing database of exosomal proteins ‘ExoCarta’. Since the ExoCarta database is compiled in gene name format, we also converted the format of our protein list (UniProt ID format) into a gene name format. While converting, we found that four proteins were excluded from the list as their gene name annotation was not completed. Therefore, the final list, which was further analyzed with ExoCarta, contains 423 proteins in the control group and 385 proteins in the prenatally cocaine-exposed group. As shown in Figure 2B, 234 CSF-EV proteins in the prenatally cocaine-exposed group and 266 CSF-EV proteins in the control group were present in the ExoCarta protein list. Interestingly, the principal component analysis showed a ‘control/prenatally cocaine-exposed’ (Figure 2C) discrimination based on relative abundances of the identified protein.

### 3.3. Ingenuity Pathway Analysis (IPA) of Proteins Identified in CSF-EVs

**Figure 3:**
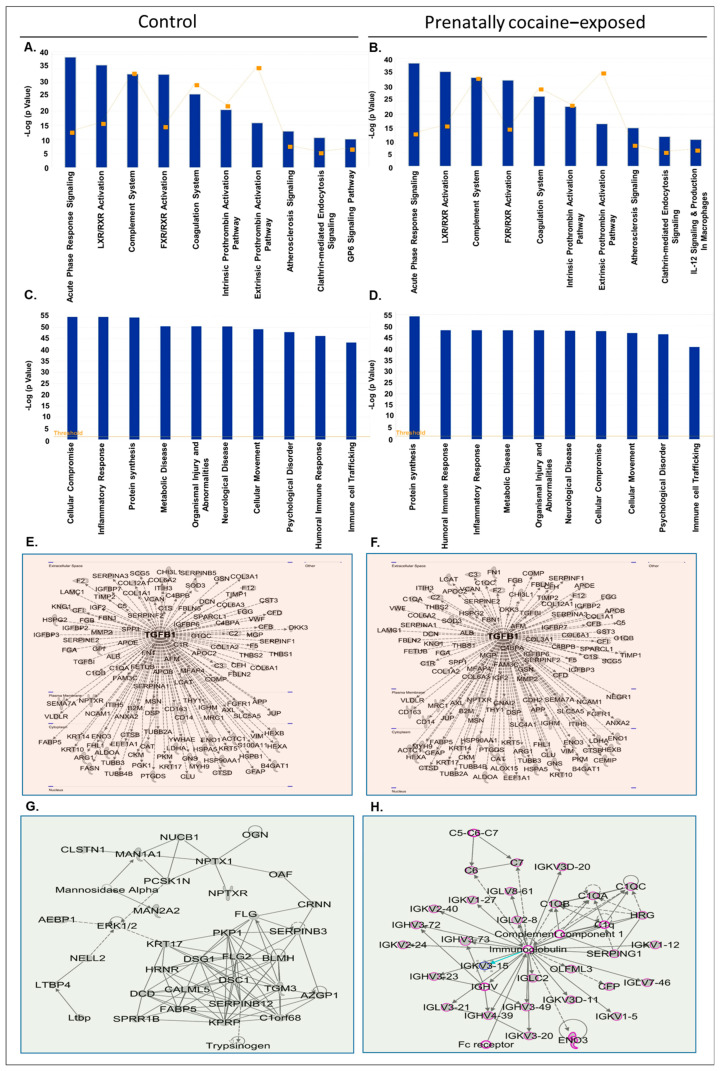
IPA analyses of proteins loaded in CSF-EVs. (**A**,**B**) Top 10 significantly enriched canonical pathways; (**C**,**D**) top 10 disease and disorders; (**E**,**F**) protein-protein interaction networks representing the upstream regulators and their targets with upstream regulator shown with bold label; and (**G**,**H**) the top enriched network in CSF-EVs in control and prenatally cocaine-exposed monkeys.

Next, we analyzed the CSF-EV proteins identified in control and prenatally cocaine- exposed monkeys with IPA software for top canonical pathways, diseases and disorders associated with the proteins, upstream regulators, and top enriched network. The list of CSF-EV proteins in control and prenatally cocaine-exposed groups is provided in Supplementary Tables S1 and S2, respectively. IPA analyses identified the top 10 canonical pathways associated with CSF-EV proteins in control (Figure 3A) and prenatally cocaine-exposed groups (Figure 3B). Acute-phase response signaling, Liver X Receptor-Retinoid X Receptor (LXR/RXR) activation, complement system, Farnesoid X receptor-Retinoid X Receptor (FXR/RXR) activation, coagulation system, intrinsic prothrombin activation pathway, extrinsic prothrombin activation pathway, atherosclerosis signaling, and clathrin-mediated endocytosis signaling were altered in both control and prenatally cocaine-exposed monkeys. Among the top 10 significantly altered pathways, Glycoprotein VI (GP6) signaling pathway was present in control monkeys, while IL-12 signaling and production in macrophages were present in prenatally cocaine-exposed monkeys.

Similarly, there was no major difference observed in the top 10 diseases and disorders associated with the CSF-EVs proteins in control (Figure 3C) and prenatally cocaine-exposed monkeys (Figure 3D). The affected diseases and disorders included cellular compromise, inflammatory response, protein synthesis, metabolic disease, organismal injury and abnormalities, neurological disease, cellular movement, psychological disorder, humoral immune response, and immune cell trafficking.

Further, the analyses of protein-protein interaction networks demonstrated transforming growth factor β1 (TGFβ1) as a common upstream regulator in CSF-EV proteins of control (Figure 3E) and prenatally cocaine-exposed monkeys (Figure 3F). Top network enriched analyses demonstrated desmoglein 1 (DSG1) and desmocolin 1 (DSC1) as key players in the CSF-EV proteins of control monkeys (Figure 3G), and complement component 1 and immunoglobulin as the key players associated with CSF-EV proteins of prenatally cocaine-exposed monkeys (Figure 3H).

### 3.4. Processing of Differentially Loaded Proteins in CSF-EVs from Control and Prenatally Cocaine-Exposed Monkeys Using IPA

**Figure 4:**
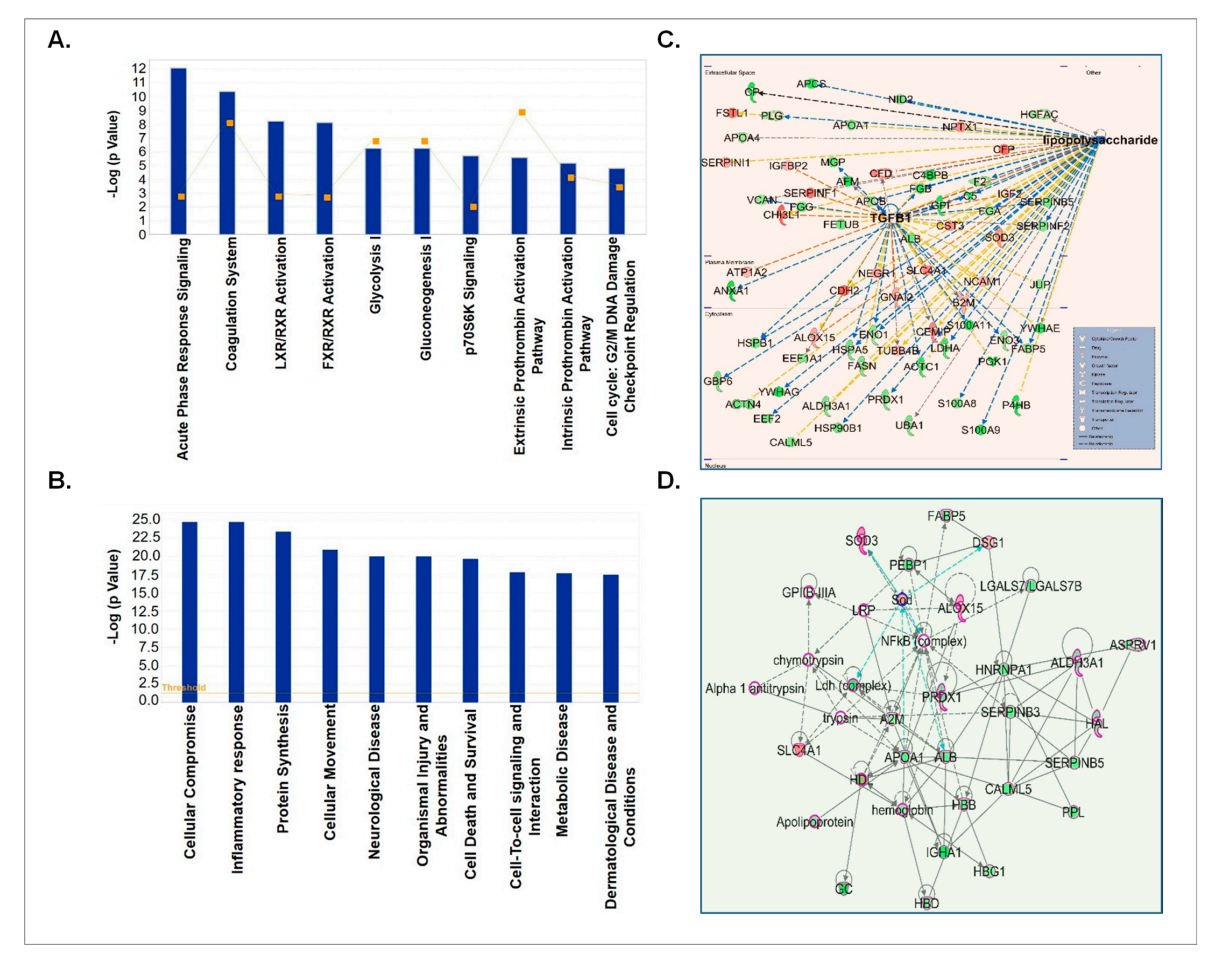
IPA analyses of differentially expressed proteins in CSF-EVs of prenatally cocaine-exposed monkeys vs. control monkeys. (**A**) Top 10 significantly enriched canonical pathways; (**B**) top 10 disease and disorders; (**C**) protein-protein interaction networks representing the upstream regulators and their targets. The upstream regulator is shown with bold label; and (**D**) top enriched network in CSF-EVs of prenatally cocaine-exposed vs control monkeys.

Next, we analyzed the differentially loaded proteins (both up- and down-regulated) in CSF-EVs between control and prenatally cocaine-exposed groups. We identified the proteins whose expression was higher (STN values ≥ 0.3; *n* = 48)) or lower (STN values ≤ −0.3; *n* = 106) in the prenatally cocaine-exposed group compared to the control group and analyzed those proteins by IPA for similar parameters as described above in Section 3.3. We first identified the top 10 canonical pathways associated with differentially loaded proteins (Figure 4A). In these pathways, acute phase response signaling (*p* = 8.23× 10^−13^), coagulation system (*p* = 4.18 × 10^−11^), LXR/RXR activation (*p* = 5.92× 10^−9^), FXR/RXR activation (*p* = 7.48 × 10^−9^), glycolysis I (*p* = 5.49 × 10^−7^), gluconeogenesis I (*p* = 5.49 × 10^−7^), p70S6K signaling (*p* = 1.96 × 10^−06^), extrinsic prothrombin activation pathway (*p* = 2.6 × 10^−6^), intrinsic prothrombin activation pathway (*p* = 6.54 × 10^−6^) and cell cycle: G2/M DNA damage checkpoint regulation (*p* = 1.56 × 10^−5^) were the most affected canonical pathways.

The top 10 diseases and disorders associated with differentially loaded proteins between the 2 groups as predicted by IPA analyses are shown in Figure 4B. This list included cellular compromise (*p* = 1.93 × 10^−25^–5.17 × 10^−5^), inflammatory responses (*p* = 1.93 × 10^−25^–8.48 × 10^−5^), protein synthesis (*p* = 4.16 × 10^−24^–1.58 × 10^−5^), cellular movement (*p* = 1.23 × 10^−21^–7.06 × 10^−5^), neurological diseases (*p* = 9.96 × 10^−21^–8.34 × 10^−5^), organismal injuries and abnormalities (*p* = 9.96 × 10^−21^–8.34 × 10^−5^), cell death and survival (*p* = 2.15 × 10^−20^–5.11 × 10^−5^), cell to cell signaling and interactions (*p* = 1.5 × 10^−18^–8.48 × 10^−5^), metabolic disease (*p* = 2.04 × 10^−18^–1.99 × 10^−5^) and dermatological disease (*p* = 3.24 × 10^−18^–8.08 × 10^−5^). Furthermore, upstream regulator analyses of these proteins predicted that lipopolysaccharide (LPS) (*p* = 6.62 × 10^−17^) and TGFβ1 (*p* = 3.79 × 10^−14^) could be the main upstream regulators (Figure 4C). Top network enrichment analyses demonstrated the nuclear factor (NF)-κB complex as one of the key players of the network (Figure 4D).

**Figure 5:**
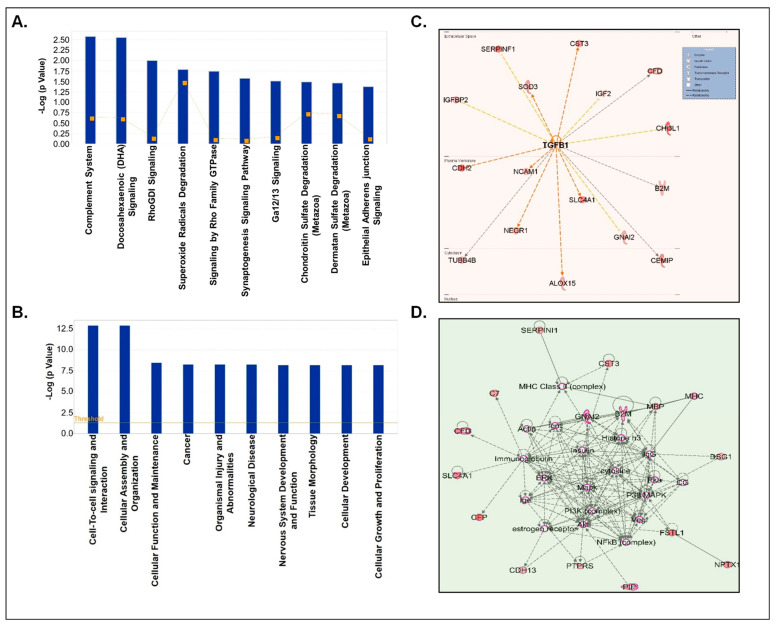
IPA analyses of upregulated proteins in CSF-EVs of prenatally cocaine-exposed monkeys vs. control monkeys. (**A**) Top 10 significantly enriched canonical pathways; (**B**) top 10 disease and disorders; (**C**) protein-protein interaction networks representing the upstream regulators and their targets. The upstream regulator is shown with bold label; and (**D**) the top enriched network in CSF-EVs of prenatally cocaine-exposed vs. control monkeys.

Next, we segregated our analyses based on up- and down-regulated proteins. First, we performed the analyses of only upregulated CSF-EV proteins in prenatally cocaine-exposed monkeys (Figure 5). The top 10 canonical pathways associated with these CSF-EV proteins are shown in Figure 5A, which included complement system (*p* = 2.67 × 10^−3^), docosahexaenoic acid (DHA) signaling (*p* = 2.82 × 10^−3^), Rho-specific guanine nucleotide dissociation inhibitor (RhoGDI) signaling (*p* = 1 × 10^−2^), superoxide radical degradation (*p* = 1.65 × 10^−2^), signaling by Ras homologous (Rho) family GTPase (*p* = 1.81 × 10^−2^), synaptogenesis signaling pathway (*p* = 2.68 × 10^−2^), chondroitin sulphate degradation (*p* = 3.26 × 10^−2^), and dermatin sulphate degradation (*p* = 3.47 × 10^−2^) and epithelial adherens junction signaling (*p* = 4.25 × 10^−2^).

The diseases and disorders associated with the proteins that were upregulated in the CSF-EVs of prenatally cocaine-exposed monkeys include cell to cell signaling and interaction (*p* = 1.22 × 10^−13^–3.6 × 10^−3^), cellular assembly and organization (*p* = 1.22 × 10^−13^–2.65 × 10^−3^), cellular function and maintenance (*p* = 3.46 × 10^−9^–2.65 × 10^−3^), cancer (*p* = 5.42 × 10^−9^–3.81 × 10^−3^), organismal injury and abnormalities (*p* = 5.42 × 10^−9^–3.81 × 10^−3^), neurological disease (*p* = 5.73 × 10^−9^–3.04 × 10^−3^), nervous system development and function (*p* = 6.7 × 10^−9^–3.6 × 10^−3^), tissue morphology (*p* = 6.7 × 10^−9^–3.79 × 10^−3^), cellular development (*p* = 6.79 × 10^−9^–3.72 × 10^−3^), and cellular growth and proliferation (*p* = 6.79 × 10^−9^–3.72 × 10^−3^) (Figure 5B). Furthermore, TGFβ1 (*p* = 9.07 × 10^−7^) emerged as the main upstream regulator of these proteins (Figure 5C). Top protein-protein interaction network enrichment analyses demonstrated that phosphoinositide 3-kinase (PI3K) and NF-κB complex among the key players of the protein network (Figure 5D).

**Figure 6:**
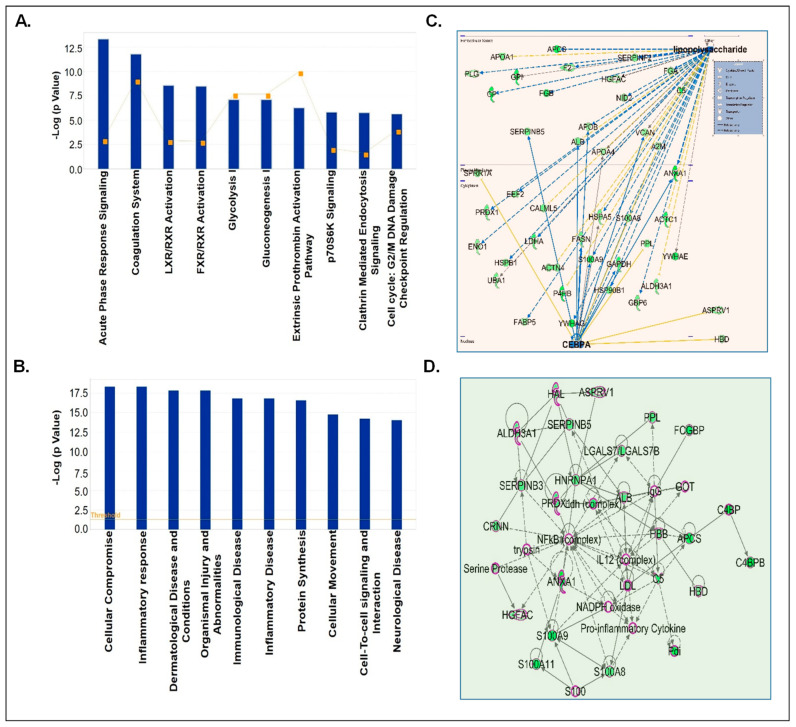
IPA analyses of significantly downregulated proteins in CSF-EVs of prenatally cocaine-exposed adult monkeys vs. control monkeys. (**A**) Top 10 significantly enriched canonical pathways; (**B**) top 10 disease and disorders; (**C**) protein-protein interaction networks representing the upstream regulators and their targets. The upstream regulators are shown with bold label; and (**D**) the top enriched network in CSF-EVs of prenatally cocaine-exposed vs. control monkeys.

Next, downregulated CSF-EV proteins in prenatally cocaine-exposed monkeys were analyzed by IPA for the top canonical pathways, diseases and disorder, upstream regulators analyses, and top enriched networks (Figure 6). IPA identified the top 10 significantly perturbed canonical pathways including acute phase response signaling (*p* = 4.74 × 10^−14^), coagulation system (*p* = 1.64 × 10^−12^), LXR/RXR activation (*p* = 2.64 × 10^−9^), FXR/RXR activation (*p* = 3.26 × 10^−9^), glycolysis I (*p* = 7.49 × 10^−8^), gluconeogenesis I (*p* = 7.49 × 10^−8^), extrinsic prothrombin activation pathway (*p* = 5.27 × 10^−7^), p70S6K signaling (*p* = 1.49 × 10^−6^), clathrin-mediated endocytosis signaling (*p* = 1.66 × 10^−6^), and cell cycle: G2/M DNA damage checkpoint regulation (*p* = 2.23 × 10^−6^) (Figure 6A).

The diseases and disorder associated with the downregulated proteins in CSF-EVs of prenatally cocaine-exposed monkeys are shown in Figure 6B and included cellular compromise (*p* = 4.5 × 10^−19^–3.67 × 10^−4^), inflammatory response (*p* = 4.5 × 10^−19^–4.34 × 10^−4^), dermatological disease and conditions (*p* = 1.44 × 10^−18^–2.63 × 10^−4^), organism injuries and abnormalities (*p* = 1.44 × 10^−18^–4.38 × 10^−4^), immunological disease (*p* = 1.49 × 10^−17^–3.42 × 10^−5^), inflammatory disease (*p* = 1.49 × 10^−17^–1.76 × 10^−4^), protein synthesis (*p* = 2.61 × 10^−17^–2.76 × 10^−4^), cellular movement (*p* = 1.78 × 10^−15^–4.34 × 10^−4^), cell to cell signaling and interaction (*p* = 6.07 × 10^−15^–3.67 × 10^−4^), and neurological disease (*p* = 9.27 × 10^−15^–4.35 × 10^−4^). Furthermore, LPS (*p* = 2.69 × 10^−14^) and CCAAT enhancer binding protein alpha (CEBPA) (*p* = 1.86 × 10^−10^) were the main upstream regulators of these proteins (Figure 6C). Lastly, top network enrichment analyses demonstrated NF-κB complex, interleukin (IL-12) and pro-inflammatory cytokines as the major protagonists of the network (Figure 6D).

## 4. Discussion

Prenatal exposure to cocaine has been reported to have long-term effects on the development and functions of the offspring’s nervous system [22,23]. Early-stage observation is required to detect these alterations, which could otherwise lead to long-term complications in the development and function of the nervous system. However, the variability and cost of sophisticated diagnostic tools, such as magnetic resonance imaging (MRI), computed tomography (CT) scan, positron emission tomography (PET), etc., make it difficult for many people. Furthermore, these techniques provide limited information of the alterations at the molecular level. To study the effects at molecular level, post-mortem brain sectioning is required. To overcome these limitations, this study focused on CSF-EVs as a potential diagnostic tool to detect the long-term consequences of prenatal cocaine exposure. Importantly, EVs derived from various biological fluids have been reported to act as potential biomarkers for various pathological as well as physiological conditions [12,17,24]. Therefore, CSF-EVs isolated from the prenatally cocaine-exposed monkeys were characterized for their surface proteins, concentration, size distribution, and proteomic analyses. We confirmed the presence of exosome-specific biomarkers (CD63, CD9, and CD81) expressed on the surface of CSF-EVs of male and female monkeys from control and prenatally cocaine-exposed group. Furthermore, the majority of the EVs were in the size range of sEV (<200 nm). Interesting, CSF-EVs average concentration (particles/mL) was 54% lower in prenatally cocaine-exposed monkeys compared with controls. This suggests a possible cocaine-induced reduction of intercellular communication between various cells in the brain. It was reported recently that cocaine administration could reduce the exosome production in microglial cells [25]. Furthermore, cocaine self-administration could alter the neuronal exosome signaling to brain cells in the nucleus accumbens in transgenic mice [26]. Therefore, through the restoration of EVs biogenesis and their regulated pathways, the adverse impact of cocaine, including its transgenerational effects, could be ameliorated. Recently, Saeedi et al. reported that antidepressant drug escitalopram treatment affects neuron-derived EV biogenesis and reverses the decreased size observed in untreated patients with major depressive disorder [27]. Earlier, Datta et al. identified several inhibitors (tipifarnib, neticonazole, climbazole, ketoconazole, and triademenol) and activators (sitafloxacin, forskolin, SB218795, fenoterol, nitrefazole, and pentetrazol) of EV biogenesis and secretion [28]. It is tempting to test some clinically approved drugs, which could activate EV biogenesis, as a potential therapeutic approach against transgenerational adverse effects of cocaine.

IPA analyses revealed no significant differences in the top 10 biological pathways and disease and disorders associated with all proteins identified in CSF-EVs in control and prenatally cocaine-exposed monkeys. For example, 9 out of the top 10 biological pathways associated with these proteins were common. However, analyses of differentially expressed proteins in CSF-EVs of prenatally cocaine-exposed monkeys in comparison to control monkeys demonstrated that prenatal cocaine exposure could be associated with neuroinflammation. The affected acute phase response usually occurs due to the hepatic response to inflammatory insult and involves hepatic production of acute-phase proteins, including serum amyloid A [29]. These proteins are extrahepatically found in the CSF of patients with neurodegenerative diseases, and their concentration is relatively higher as compared to controls [30]. Although the cellular origin of serum amyloid A is unclear, Barbierato et al. reported that pro-inflammatory cytokine tumor necrosis factor (TNF)-α and LPS increase its mRNA expression in cultured rat microglia [31]. LPS was also the predicted upstream regulator in IPA analyses. TGFβ1 is another predicted upstream regulator; it protects neurons against various toxins and injurious agents, both in vitro and in vivo [32]. TGFβ1 signaling increases with age; astrocytes and activated microglia and macrophages are the main cell types that undergo increased TGFβ1 signaling in response to the post-stroke increase in TGFβ1 [33]. Other affected biological pathways include coagulation system, LXR/RXR activation, extrinsic prothrombin activation pathway, glycolysis I, gluconeogenesis I, and intrinsic prothrombin activation pathway. Studies have reported proteins associated with some of these altered signals in CSF of Alzheimer’s disease (AD) patients [34]. The top 10 predicted diseases and disorders associated included inflammatory responses and neurological diseases. Prediction of NF-κB complex in the top network enrichment analyses also demonstrated that prenatal cocaine exposure could lead to inflammation. NF-κB plays an important role in host innate immune responses. It has also been reported that substance use and abuse can induce NF-κB activity and cytokine expression in the human brain [35,36]. Overall, the present results suggest that prenatal cocaine exposure could be associated with neuronal inflammatory responses in adulthood.

Analyses of upregulated proteins in CSF-EVs from prenatally cocaine-exposed monkeys identified association with DHA signaling, superoxide radical degradation, and synaptogenesis signaling pathway, among others. DHA is an omega-3 long-chain polyunsaturated fatty acid relevant for brain function. During gestation and breastfeeding, most of the DHA found in the brain is supplied from the mother [37]. The superoxide radical has a well-known role in the inflammatory process and oxidative stress, which is involved in the progression of neurodegenerative diseases. It has been suggested that the damage resulting from the reinforcing effects of cocaine (and the continued use of cocaine) is associated with increased reactive oxygen species (ROS) production [38,39]. Synaptogenesis signaling is involved in a long developmental process of synapsis formation, maintenance, and activity-dependent synapse refinement, and is important for the establishment of the neuronal network and the precision of brain circuitry. Recently, cocaine was reported to alter synaptogenesis [40]. Analyses of downregulated CSF-EVs proteins in prenatally cocaine-exposed monkeys demonstrated that the clathrin-mediated endocytosis signaling pathway is affected in the prenatally cocaine-exposed monkeys. Clathrin-mediated endocytosis has a role in exosome internalization [41], suggesting that exosome-related pathways may be altered. The diseases and disorders associated with these proteins included inflammatory response and neurological disease. LPS and CEBPA were the main upstream regulators. Our findings are consistent with Fries et al., who demonstrated that anhedonia in cocaine use disorder was associated with differential expression of the inflammatory gene, including CEBPA in peripheral blood cells [42]. NF-κB complex and IL-12 are the predicted protagonists of the network by top network enrichment analyses. Overall, alterations in these signaling pathways have well-defined roles in brain development and neurodegeneration. Therefore, their alteration may be associated with an increased tendency of prenatally cocaine-exposed individuals to potentially develop CNS-related disorders.

Outcomes of the present study are important in terms of identifying several deregulated pathways in the CSF of prenatally cocaine-exposed monkeys; however, there are a few major limitations of this study. For example, we employed a precipitation-based single methodology to isolate EVs for maximum recovery as CSF is relatively a poor source of EVs. While our NTA data suggested an enrichment of sEV, an inherent limitation of this procedure is the co-precipitation of free proteins and larger EVs, which cannot be ruled out in our preparation. In addition, MS data revealed a low abundance and high heterogeneity in protein loading between samples, even within samples from the same group. Furthermore, we cannot rule out the potential adverse effects of CSF storage on the quality and quantity of EVs, though in most published studies, similar sample storage conditions have been reported, and both prenatal cocaine and control samples were handled in identical manners. Lastly, it is important to validate the key findings of the MS data by other tools such as Western blotting or ELISA.

In conclusion, we have successfully isolated and characterized EVs from CSF of prenatally cocaine-exposed monkeys to identify biomarker/s of potential long-term adverse effects. EVs exhibited characteristics of sEV size and exosomal biomarkers. The differentially expressed proteins in CSF-EVs demonstrated that prenatal cocaine exposure could be associated with CNS-related disorders. Our study indicates that the CSF-EVs could offer potential biomarkers for the diagnosis of nervous system-related disorders associated with prenatal cocaine exposure. However, further, well-controlled neuropsychological studies are needed to correlate these EV biomarkers with cognition, memory, and other executive functions.

## Data Availability

Study-specific material can be provided upon written request to the corresponding authors. There is no restriction on the availability of any data.

## Supplementary Materials

The following supporting information can be downloaded at: https://www.mdpi.com/article/10.3390/biom12040510/s1, Table S1: List of proteins identified (416) in CSF-derived EVs in control monkeys. Table S2: List of proteins identified (379) in CSF-derived EVs in prenatally cocaine-exposed monkeys.
