# Supplementary material for: Mass Spectrometry-Based Proteome Profiling of Extracellular Vesicles Derived from the Cerebrospinal Fluid of Adult Rhesus Monkeys Exposed to Cocaine throughout Gestation
Source: Biomolecules. 2022 Mar 28;12(4):510. doi: 10.3390/biom12040510 (PMC9026784; doi:10.3390/biom12040510)
Supplement: Supplementary file 1 [file biomolecules-12-00510-s001.zip › biomolecules-1550934-supplementary.pptx]

## Slide 1
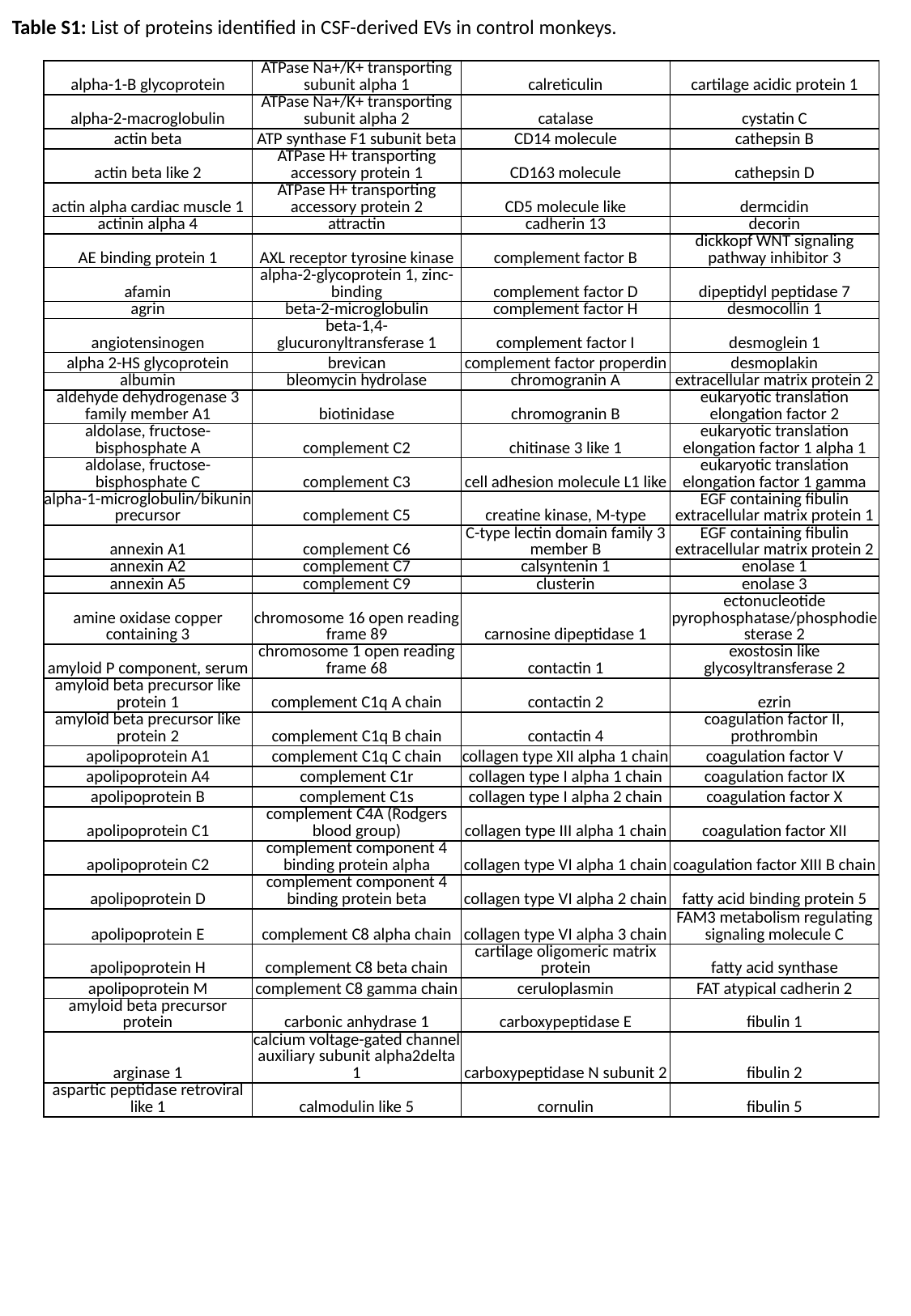

Table S1: List of proteins identified in CSF-derived EVs in control monkeys.
| alpha-1-B glycoprotein | ATPase Na+/K+ transporting subunit alpha 1 | calreticulin | cartilage acidic protein 1 |
| --- | --- | --- | --- |
| alpha-2-macroglobulin | ATPase Na+/K+ transporting subunit alpha 2 | catalase | cystatin C |
| actin beta | ATP synthase F1 subunit beta | CD14 molecule | cathepsin B |
| actin beta like 2 | ATPase H+ transporting accessory protein 1 | CD163 molecule | cathepsin D |
| actin alpha cardiac muscle 1 | ATPase H+ transporting accessory protein 2 | CD5 molecule like | dermcidin |
| actinin alpha 4 | attractin | cadherin 13 | decorin |
| AE binding protein 1 | AXL receptor tyrosine kinase | complement factor B | dickkopf WNT signaling pathway inhibitor 3 |
| afamin | alpha-2-glycoprotein 1, zinc-binding | complement factor D | dipeptidyl peptidase 7 |
| agrin | beta-2-microglobulin | complement factor H | desmocollin 1 |
| angiotensinogen | beta-1,4-glucuronyltransferase 1 | complement factor I | desmoglein 1 |
| alpha 2-HS glycoprotein | brevican | complement factor properdin | desmoplakin |
| albumin | bleomycin hydrolase | chromogranin A | extracellular matrix protein 2 |
| aldehyde dehydrogenase 3 family member A1 | biotinidase | chromogranin B | eukaryotic translation elongation factor 2 |
| aldolase, fructose-bisphosphate A | complement C2 | chitinase 3 like 1 | eukaryotic translation elongation factor 1 alpha 1 |
| aldolase, fructose-bisphosphate C | complement C3 | cell adhesion molecule L1 like | eukaryotic translation elongation factor 1 gamma |
| alpha-1-microglobulin/bikunin precursor | complement C5 | creatine kinase, M-type | EGF containing fibulin extracellular matrix protein 1 |
| annexin A1 | complement C6 | C-type lectin domain family 3 member B | EGF containing fibulin extracellular matrix protein 2 |
| annexin A2 | complement C7 | calsyntenin 1 | enolase 1 |
| annexin A5 | complement C9 | clusterin | enolase 3 |
| amine oxidase copper containing 3 | chromosome 16 open reading frame 89 | carnosine dipeptidase 1 | ectonucleotide pyrophosphatase/phosphodiesterase 2 |
| amyloid P component, serum | chromosome 1 open reading frame 68 | contactin 1 | exostosin like glycosyltransferase 2 |
| amyloid beta precursor like protein 1 | complement C1q A chain | contactin 2 | ezrin |
| amyloid beta precursor like protein 2 | complement C1q B chain | contactin 4 | coagulation factor II, prothrombin |
| apolipoprotein A1 | complement C1q C chain | collagen type XII alpha 1 chain | coagulation factor V |
| apolipoprotein A4 | complement C1r | collagen type I alpha 1 chain | coagulation factor IX |
| apolipoprotein B | complement C1s | collagen type I alpha 2 chain | coagulation factor X |
| apolipoprotein C1 | complement C4A (Rodgers blood group) | collagen type III alpha 1 chain | coagulation factor XII |
| apolipoprotein C2 | complement component 4 binding protein alpha | collagen type VI alpha 1 chain | coagulation factor XIII B chain |
| apolipoprotein D | complement component 4 binding protein beta | collagen type VI alpha 2 chain | fatty acid binding protein 5 |
| apolipoprotein E | complement C8 alpha chain | collagen type VI alpha 3 chain | FAM3 metabolism regulating signaling molecule C |
| apolipoprotein H | complement C8 beta chain | cartilage oligomeric matrix protein | fatty acid synthase |
| apolipoprotein M | complement C8 gamma chain | ceruloplasmin | FAT atypical cadherin 2 |
| amyloid beta precursor protein | carbonic anhydrase 1 | carboxypeptidase E | fibulin 1 |
| arginase 1 | calcium voltage-gated channel auxiliary subunit alpha2delta 1 | carboxypeptidase N subunit 2 | fibulin 2 |
| aspartic peptidase retroviral like 1 | calmodulin like 5 | cornulin | fibulin 5 |

## Slide 2
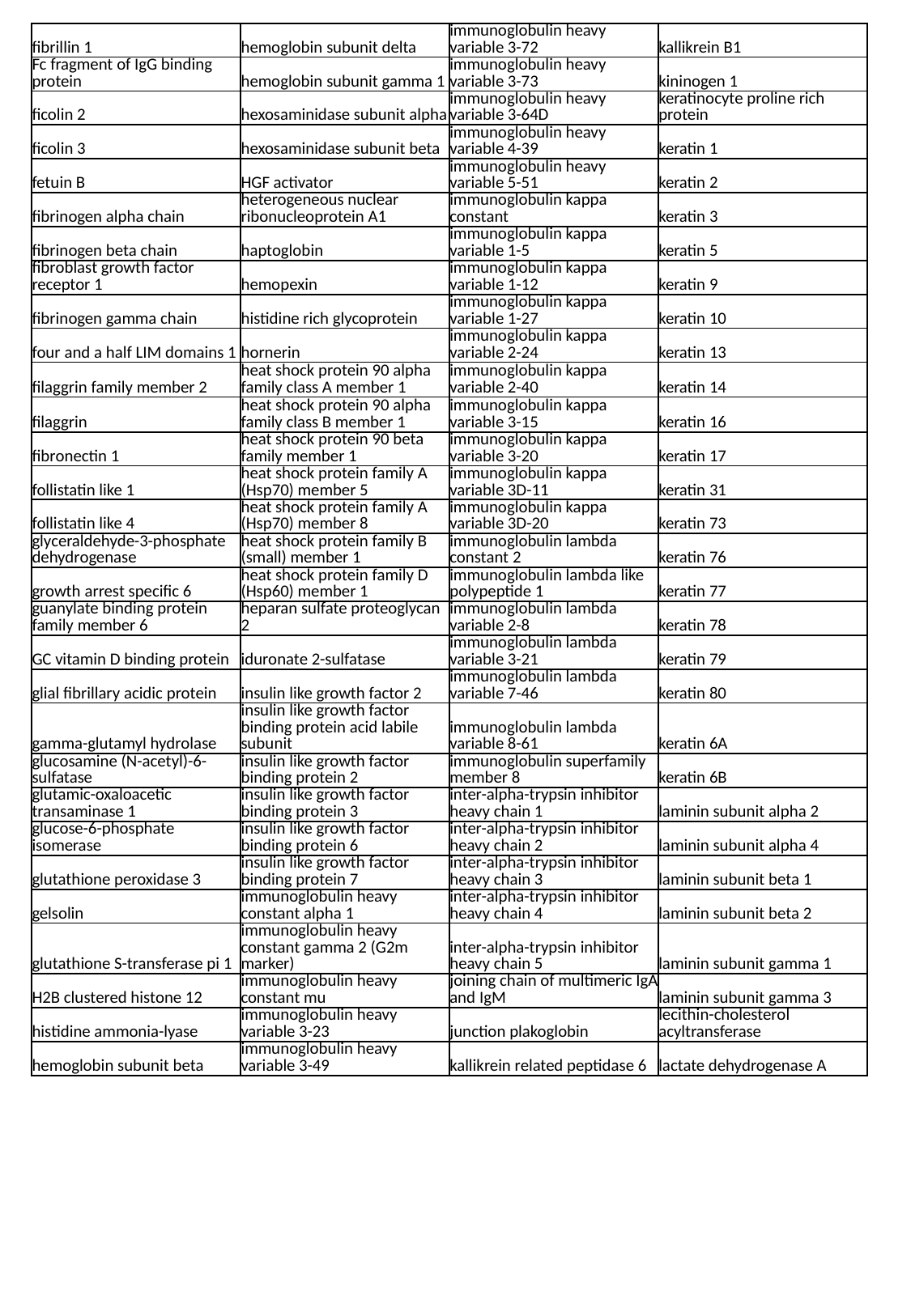

| fibrillin 1 | hemoglobin subunit delta | immunoglobulin heavy variable 3-72 | kallikrein B1 |
| --- | --- | --- | --- |
| Fc fragment of IgG binding protein | hemoglobin subunit gamma 1 | immunoglobulin heavy variable 3-73 | kininogen 1 |
| ficolin 2 | hexosaminidase subunit alpha | immunoglobulin heavy variable 3-64D | keratinocyte proline rich protein |
| ficolin 3 | hexosaminidase subunit beta | immunoglobulin heavy variable 4-39 | keratin 1 |
| fetuin B | HGF activator | immunoglobulin heavy variable 5-51 | keratin 2 |
| fibrinogen alpha chain | heterogeneous nuclear ribonucleoprotein A1 | immunoglobulin kappa constant | keratin 3 |
| fibrinogen beta chain | haptoglobin | immunoglobulin kappa variable 1-5 | keratin 5 |
| fibroblast growth factor receptor 1 | hemopexin | immunoglobulin kappa variable 1-12 | keratin 9 |
| fibrinogen gamma chain | histidine rich glycoprotein | immunoglobulin kappa variable 1-27 | keratin 10 |
| four and a half LIM domains 1 | hornerin | immunoglobulin kappa variable 2-24 | keratin 13 |
| filaggrin family member 2 | heat shock protein 90 alpha family class A member 1 | immunoglobulin kappa variable 2-40 | keratin 14 |
| filaggrin | heat shock protein 90 alpha family class B member 1 | immunoglobulin kappa variable 3-15 | keratin 16 |
| fibronectin 1 | heat shock protein 90 beta family member 1 | immunoglobulin kappa variable 3-20 | keratin 17 |
| follistatin like 1 | heat shock protein family A (Hsp70) member 5 | immunoglobulin kappa variable 3D-11 | keratin 31 |
| follistatin like 4 | heat shock protein family A (Hsp70) member 8 | immunoglobulin kappa variable 3D-20 | keratin 73 |
| glyceraldehyde-3-phosphate dehydrogenase | heat shock protein family B (small) member 1 | immunoglobulin lambda constant 2 | keratin 76 |
| growth arrest specific 6 | heat shock protein family D (Hsp60) member 1 | immunoglobulin lambda like polypeptide 1 | keratin 77 |
| guanylate binding protein family member 6 | heparan sulfate proteoglycan 2 | immunoglobulin lambda variable 2-8 | keratin 78 |
| GC vitamin D binding protein | iduronate 2-sulfatase | immunoglobulin lambda variable 3-21 | keratin 79 |
| glial fibrillary acidic protein | insulin like growth factor 2 | immunoglobulin lambda variable 7-46 | keratin 80 |
| gamma-glutamyl hydrolase | insulin like growth factor binding protein acid labile subunit | immunoglobulin lambda variable 8-61 | keratin 6A |
| glucosamine (N-acetyl)-6-sulfatase | insulin like growth factor binding protein 2 | immunoglobulin superfamily member 8 | keratin 6B |
| glutamic-oxaloacetic transaminase 1 | insulin like growth factor binding protein 3 | inter-alpha-trypsin inhibitor heavy chain 1 | laminin subunit alpha 2 |
| glucose-6-phosphate isomerase | insulin like growth factor binding protein 6 | inter-alpha-trypsin inhibitor heavy chain 2 | laminin subunit alpha 4 |
| glutathione peroxidase 3 | insulin like growth factor binding protein 7 | inter-alpha-trypsin inhibitor heavy chain 3 | laminin subunit beta 1 |
| gelsolin | immunoglobulin heavy constant alpha 1 | inter-alpha-trypsin inhibitor heavy chain 4 | laminin subunit beta 2 |
| glutathione S-transferase pi 1 | immunoglobulin heavy constant gamma 2 (G2m marker) | inter-alpha-trypsin inhibitor heavy chain 5 | laminin subunit gamma 1 |
| H2B clustered histone 12 | immunoglobulin heavy constant mu | joining chain of multimeric IgA and IgM | laminin subunit gamma 3 |
| histidine ammonia-lyase | immunoglobulin heavy variable 3-23 | junction plakoglobin | lecithin-cholesterol acyltransferase |
| hemoglobin subunit beta | immunoglobulin heavy variable 3-49 | kallikrein related peptidase 6 | lactate dehydrogenase A |

## Slide 3
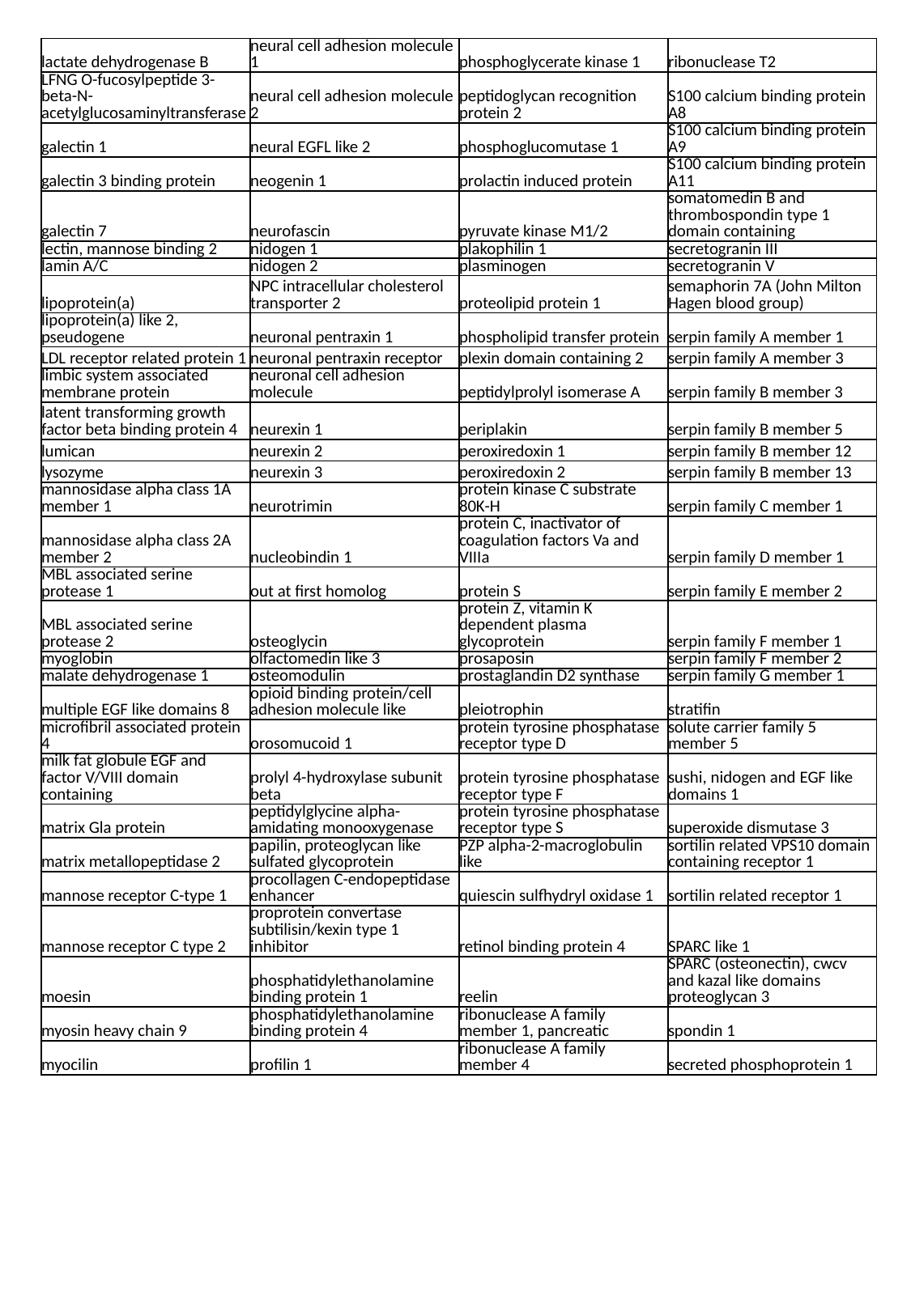

| lactate dehydrogenase B | neural cell adhesion molecule 1 | phosphoglycerate kinase 1 | ribonuclease T2 |
| --- | --- | --- | --- |
| LFNG O-fucosylpeptide 3-beta-N-acetylglucosaminyltransferase | neural cell adhesion molecule 2 | peptidoglycan recognition protein 2 | S100 calcium binding protein A8 |
| galectin 1 | neural EGFL like 2 | phosphoglucomutase 1 | S100 calcium binding protein A9 |
| galectin 3 binding protein | neogenin 1 | prolactin induced protein | S100 calcium binding protein A11 |
| galectin 7 | neurofascin | pyruvate kinase M1/2 | somatomedin B and thrombospondin type 1 domain containing |
| lectin, mannose binding 2 | nidogen 1 | plakophilin 1 | secretogranin III |
| lamin A/C | nidogen 2 | plasminogen | secretogranin V |
| lipoprotein(a) | NPC intracellular cholesterol transporter 2 | proteolipid protein 1 | semaphorin 7A (John Milton Hagen blood group) |
| lipoprotein(a) like 2, pseudogene | neuronal pentraxin 1 | phospholipid transfer protein | serpin family A member 1 |
| LDL receptor related protein 1 | neuronal pentraxin receptor | plexin domain containing 2 | serpin family A member 3 |
| limbic system associated membrane protein | neuronal cell adhesion molecule | peptidylprolyl isomerase A | serpin family B member 3 |
| latent transforming growth factor beta binding protein 4 | neurexin 1 | periplakin | serpin family B member 5 |
| lumican | neurexin 2 | peroxiredoxin 1 | serpin family B member 12 |
| lysozyme | neurexin 3 | peroxiredoxin 2 | serpin family B member 13 |
| mannosidase alpha class 1A member 1 | neurotrimin | protein kinase C substrate 80K-H | serpin family C member 1 |
| mannosidase alpha class 2A member 2 | nucleobindin 1 | protein C, inactivator of coagulation factors Va and VIIIa | serpin family D member 1 |
| MBL associated serine protease 1 | out at first homolog | protein S | serpin family E member 2 |
| MBL associated serine protease 2 | osteoglycin | protein Z, vitamin K dependent plasma glycoprotein | serpin family F member 1 |
| myoglobin | olfactomedin like 3 | prosaposin | serpin family F member 2 |
| malate dehydrogenase 1 | osteomodulin | prostaglandin D2 synthase | serpin family G member 1 |
| multiple EGF like domains 8 | opioid binding protein/cell adhesion molecule like | pleiotrophin | stratifin |
| microfibril associated protein 4 | orosomucoid 1 | protein tyrosine phosphatase receptor type D | solute carrier family 5 member 5 |
| milk fat globule EGF and factor V/VIII domain containing | prolyl 4-hydroxylase subunit beta | protein tyrosine phosphatase receptor type F | sushi, nidogen and EGF like domains 1 |
| matrix Gla protein | peptidylglycine alpha-amidating monooxygenase | protein tyrosine phosphatase receptor type S | superoxide dismutase 3 |
| matrix metallopeptidase 2 | papilin, proteoglycan like sulfated glycoprotein | PZP alpha-2-macroglobulin like | sortilin related VPS10 domain containing receptor 1 |
| mannose receptor C-type 1 | procollagen C-endopeptidase enhancer | quiescin sulfhydryl oxidase 1 | sortilin related receptor 1 |
| mannose receptor C type 2 | proprotein convertase subtilisin/kexin type 1 inhibitor | retinol binding protein 4 | SPARC like 1 |
| moesin | phosphatidylethanolamine binding protein 1 | reelin | SPARC (osteonectin), cwcv and kazal like domains proteoglycan 3 |
| myosin heavy chain 9 | phosphatidylethanolamine binding protein 4 | ribonuclease A family member 1, pancreatic | spondin 1 |
| myocilin | profilin 1 | ribonuclease A family member 4 | secreted phosphoprotein 1 |

## Slide 4
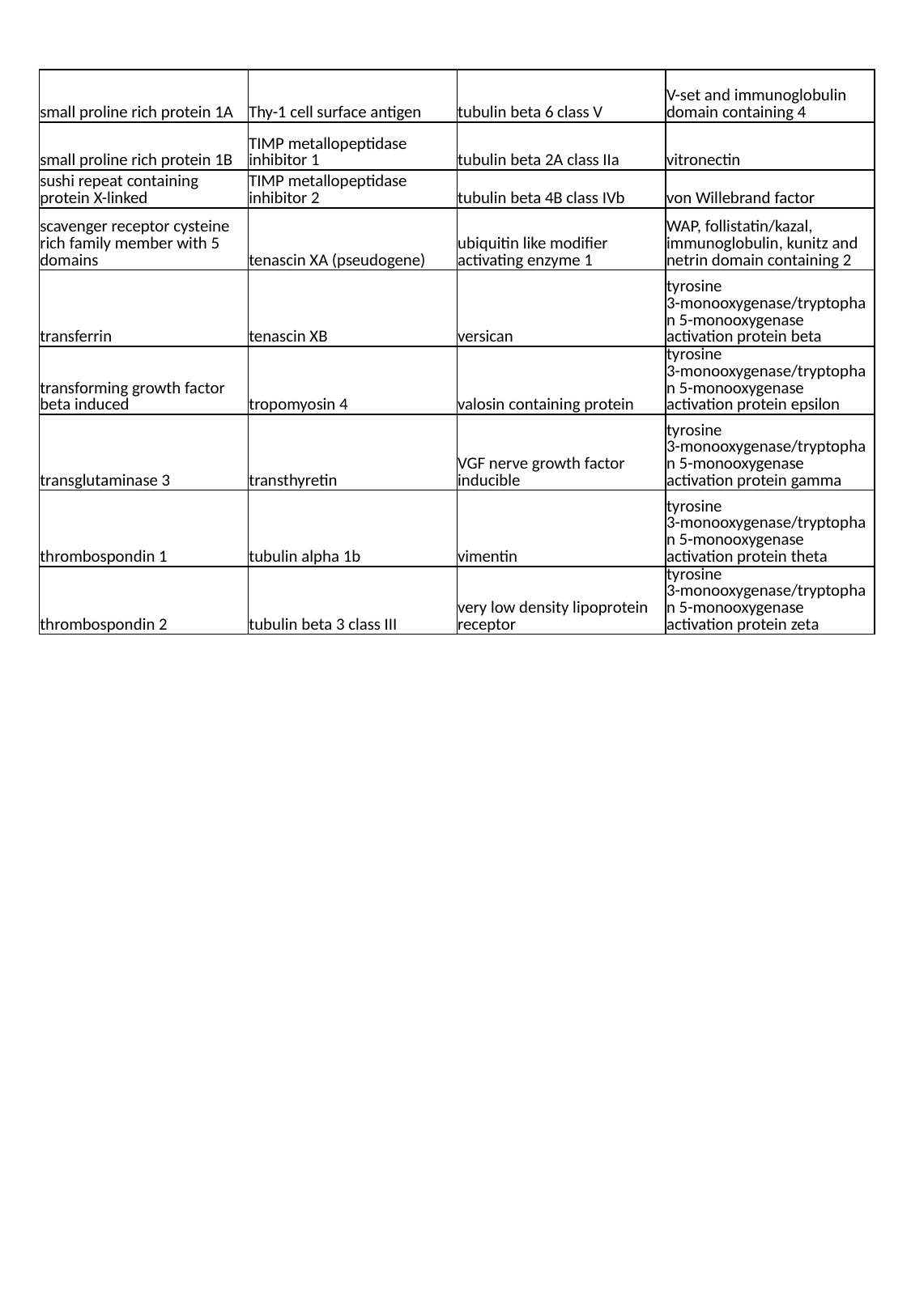

| small proline rich protein 1A | Thy-1 cell surface antigen | tubulin beta 6 class V | V-set and immunoglobulin domain containing 4 |
| --- | --- | --- | --- |
| small proline rich protein 1B | TIMP metallopeptidase inhibitor 1 | tubulin beta 2A class IIa | vitronectin |
| sushi repeat containing protein X-linked | TIMP metallopeptidase inhibitor 2 | tubulin beta 4B class IVb | von Willebrand factor |
| scavenger receptor cysteine rich family member with 5 domains | tenascin XA (pseudogene) | ubiquitin like modifier activating enzyme 1 | WAP, follistatin/kazal, immunoglobulin, kunitz and netrin domain containing 2 |
| transferrin | tenascin XB | versican | tyrosine 3-monooxygenase/tryptophan 5-monooxygenase activation protein beta |
| transforming growth factor beta induced | tropomyosin 4 | valosin containing protein | tyrosine 3-monooxygenase/tryptophan 5-monooxygenase activation protein epsilon |
| transglutaminase 3 | transthyretin | VGF nerve growth factor inducible | tyrosine 3-monooxygenase/tryptophan 5-monooxygenase activation protein gamma |
| thrombospondin 1 | tubulin alpha 1b | vimentin | tyrosine 3-monooxygenase/tryptophan 5-monooxygenase activation protein theta |
| thrombospondin 2 | tubulin beta 3 class III | very low density lipoprotein receptor | tyrosine 3-monooxygenase/tryptophan 5-monooxygenase activation protein zeta |

## Slide 5
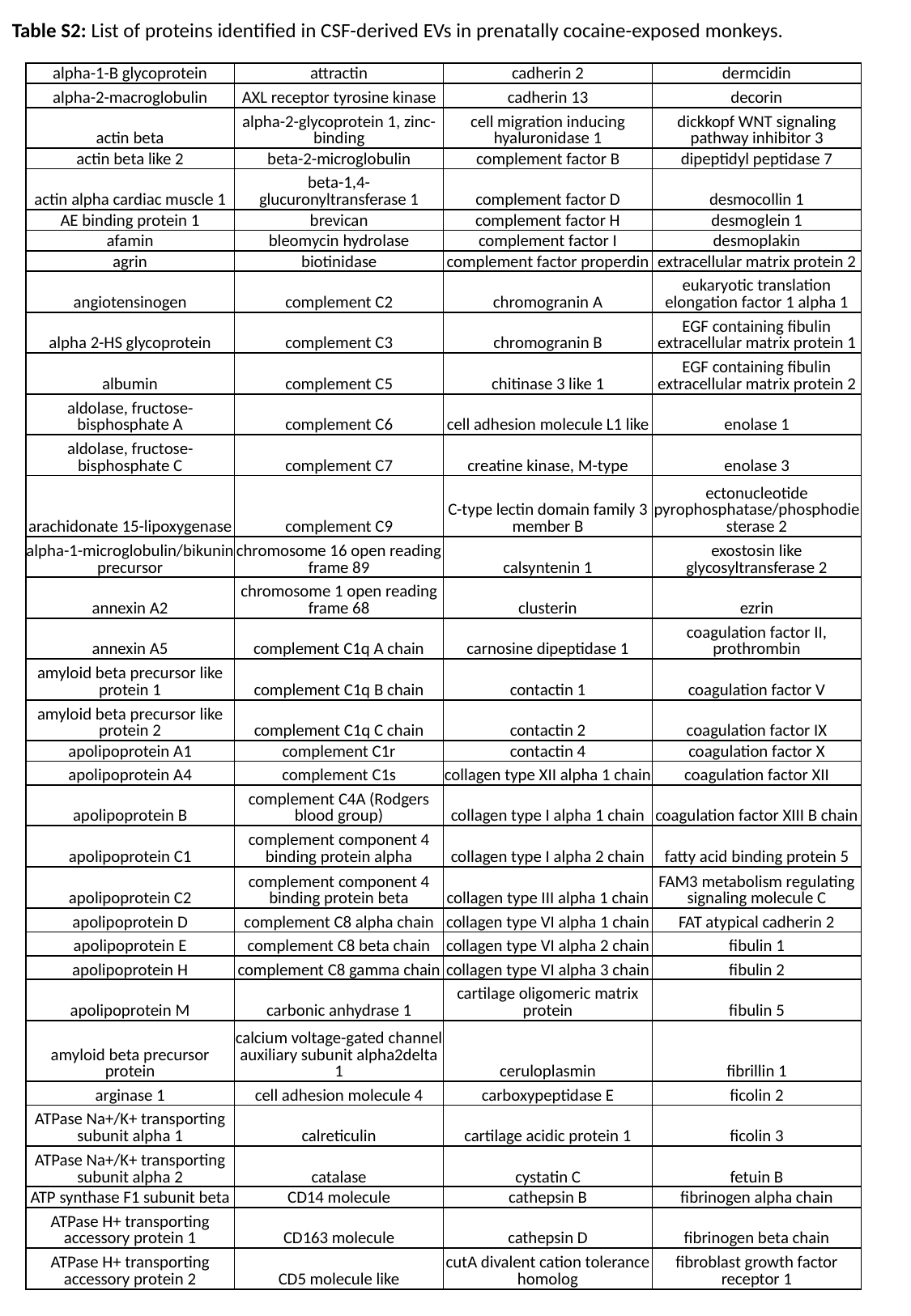

Table S2: List of proteins identified in CSF-derived EVs in prenatally cocaine-exposed monkeys.
| alpha-1-B glycoprotein | attractin | cadherin 2 | dermcidin |
| --- | --- | --- | --- |
| alpha-2-macroglobulin | AXL receptor tyrosine kinase | cadherin 13 | decorin |
| actin beta | alpha-2-glycoprotein 1, zinc-binding | cell migration inducing hyaluronidase 1 | dickkopf WNT signaling pathway inhibitor 3 |
| actin beta like 2 | beta-2-microglobulin | complement factor B | dipeptidyl peptidase 7 |
| actin alpha cardiac muscle 1 | beta-1,4-glucuronyltransferase 1 | complement factor D | desmocollin 1 |
| AE binding protein 1 | brevican | complement factor H | desmoglein 1 |
| afamin | bleomycin hydrolase | complement factor I | desmoplakin |
| agrin | biotinidase | complement factor properdin | extracellular matrix protein 2 |
| angiotensinogen | complement C2 | chromogranin A | eukaryotic translation elongation factor 1 alpha 1 |
| alpha 2-HS glycoprotein | complement C3 | chromogranin B | EGF containing fibulin extracellular matrix protein 1 |
| albumin | complement C5 | chitinase 3 like 1 | EGF containing fibulin extracellular matrix protein 2 |
| aldolase, fructose-bisphosphate A | complement C6 | cell adhesion molecule L1 like | enolase 1 |
| aldolase, fructose-bisphosphate C | complement C7 | creatine kinase, M-type | enolase 3 |
| arachidonate 15-lipoxygenase | complement C9 | C-type lectin domain family 3 member B | ectonucleotide pyrophosphatase/phosphodiesterase 2 |
| alpha-1-microglobulin/bikunin precursor | chromosome 16 open reading frame 89 | calsyntenin 1 | exostosin like glycosyltransferase 2 |
| annexin A2 | chromosome 1 open reading frame 68 | clusterin | ezrin |
| annexin A5 | complement C1q A chain | carnosine dipeptidase 1 | coagulation factor II, prothrombin |
| amyloid beta precursor like protein 1 | complement C1q B chain | contactin 1 | coagulation factor V |
| amyloid beta precursor like protein 2 | complement C1q C chain | contactin 2 | coagulation factor IX |
| apolipoprotein A1 | complement C1r | contactin 4 | coagulation factor X |
| apolipoprotein A4 | complement C1s | collagen type XII alpha 1 chain | coagulation factor XII |
| apolipoprotein B | complement C4A (Rodgers blood group) | collagen type I alpha 1 chain | coagulation factor XIII B chain |
| apolipoprotein C1 | complement component 4 binding protein alpha | collagen type I alpha 2 chain | fatty acid binding protein 5 |
| apolipoprotein C2 | complement component 4 binding protein beta | collagen type III alpha 1 chain | FAM3 metabolism regulating signaling molecule C |
| apolipoprotein D | complement C8 alpha chain | collagen type VI alpha 1 chain | FAT atypical cadherin 2 |
| apolipoprotein E | complement C8 beta chain | collagen type VI alpha 2 chain | fibulin 1 |
| apolipoprotein H | complement C8 gamma chain | collagen type VI alpha 3 chain | fibulin 2 |
| apolipoprotein M | carbonic anhydrase 1 | cartilage oligomeric matrix protein | fibulin 5 |
| amyloid beta precursor protein | calcium voltage-gated channel auxiliary subunit alpha2delta 1 | ceruloplasmin | fibrillin 1 |
| arginase 1 | cell adhesion molecule 4 | carboxypeptidase E | ficolin 2 |
| ATPase Na+/K+ transporting subunit alpha 1 | calreticulin | cartilage acidic protein 1 | ficolin 3 |
| ATPase Na+/K+ transporting subunit alpha 2 | catalase | cystatin C | fetuin B |
| ATP synthase F1 subunit beta | CD14 molecule | cathepsin B | fibrinogen alpha chain |
| ATPase H+ transporting accessory protein 1 | CD163 molecule | cathepsin D | fibrinogen beta chain |
| ATPase H+ transporting accessory protein 2 | CD5 molecule like | cutA divalent cation tolerance homolog | fibroblast growth factor receptor 1 |

## Slide 6
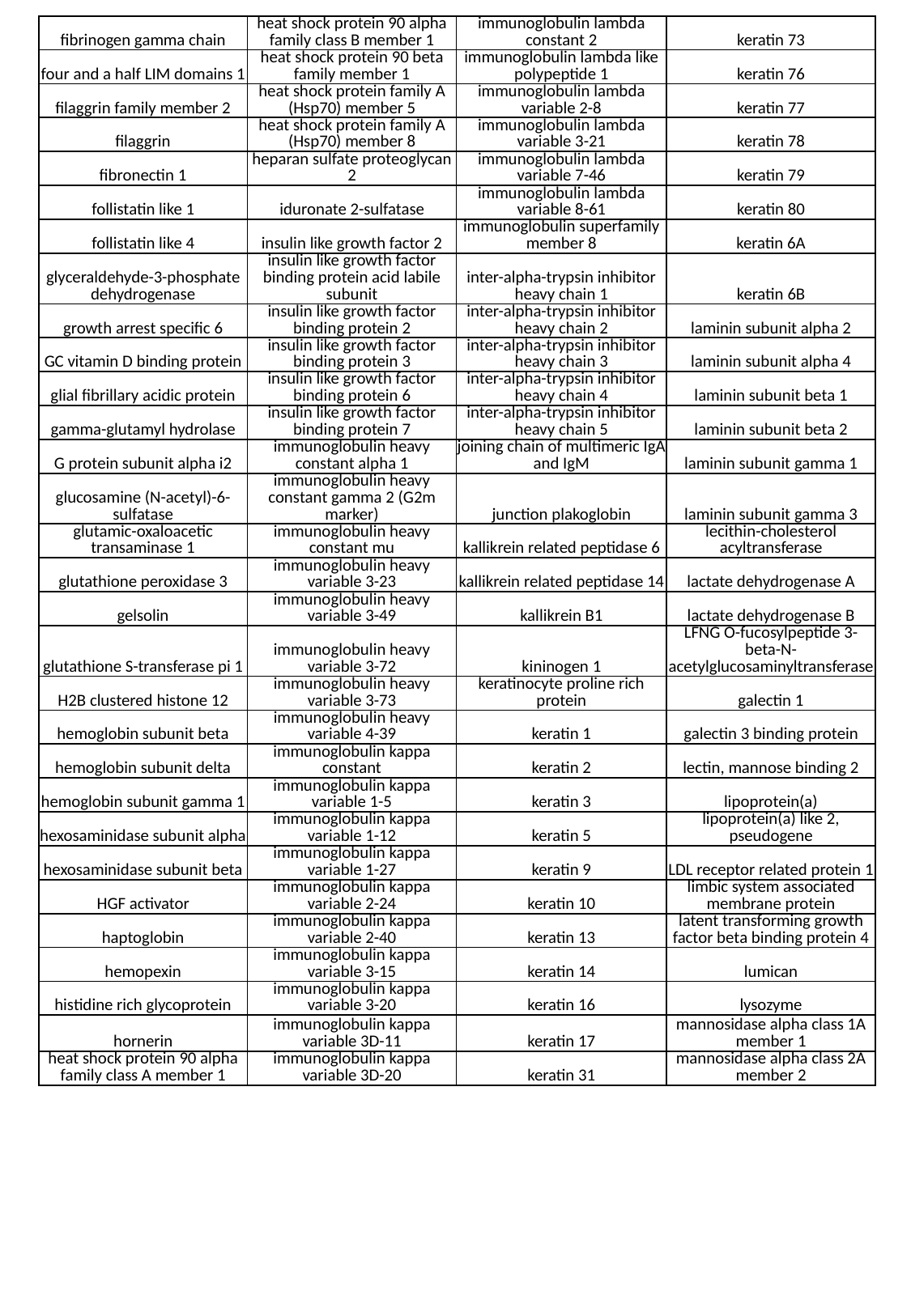

| fibrinogen gamma chain | heat shock protein 90 alpha family class B member 1 | immunoglobulin lambda constant 2 | keratin 73 |
| --- | --- | --- | --- |
| four and a half LIM domains 1 | heat shock protein 90 beta family member 1 | immunoglobulin lambda like polypeptide 1 | keratin 76 |
| filaggrin family member 2 | heat shock protein family A (Hsp70) member 5 | immunoglobulin lambda variable 2-8 | keratin 77 |
| filaggrin | heat shock protein family A (Hsp70) member 8 | immunoglobulin lambda variable 3-21 | keratin 78 |
| fibronectin 1 | heparan sulfate proteoglycan 2 | immunoglobulin lambda variable 7-46 | keratin 79 |
| follistatin like 1 | iduronate 2-sulfatase | immunoglobulin lambda variable 8-61 | keratin 80 |
| follistatin like 4 | insulin like growth factor 2 | immunoglobulin superfamily member 8 | keratin 6A |
| glyceraldehyde-3-phosphate dehydrogenase | insulin like growth factor binding protein acid labile subunit | inter-alpha-trypsin inhibitor heavy chain 1 | keratin 6B |
| growth arrest specific 6 | insulin like growth factor binding protein 2 | inter-alpha-trypsin inhibitor heavy chain 2 | laminin subunit alpha 2 |
| GC vitamin D binding protein | insulin like growth factor binding protein 3 | inter-alpha-trypsin inhibitor heavy chain 3 | laminin subunit alpha 4 |
| glial fibrillary acidic protein | insulin like growth factor binding protein 6 | inter-alpha-trypsin inhibitor heavy chain 4 | laminin subunit beta 1 |
| gamma-glutamyl hydrolase | insulin like growth factor binding protein 7 | inter-alpha-trypsin inhibitor heavy chain 5 | laminin subunit beta 2 |
| G protein subunit alpha i2 | immunoglobulin heavy constant alpha 1 | joining chain of multimeric IgA and IgM | laminin subunit gamma 1 |
| glucosamine (N-acetyl)-6-sulfatase | immunoglobulin heavy constant gamma 2 (G2m marker) | junction plakoglobin | laminin subunit gamma 3 |
| glutamic-oxaloacetic transaminase 1 | immunoglobulin heavy constant mu | kallikrein related peptidase 6 | lecithin-cholesterol acyltransferase |
| glutathione peroxidase 3 | immunoglobulin heavy variable 3-23 | kallikrein related peptidase 14 | lactate dehydrogenase A |
| gelsolin | immunoglobulin heavy variable 3-49 | kallikrein B1 | lactate dehydrogenase B |
| glutathione S-transferase pi 1 | immunoglobulin heavy variable 3-72 | kininogen 1 | LFNG O-fucosylpeptide 3-beta-N-acetylglucosaminyltransferase |
| H2B clustered histone 12 | immunoglobulin heavy variable 3-73 | keratinocyte proline rich protein | galectin 1 |
| hemoglobin subunit beta | immunoglobulin heavy variable 4-39 | keratin 1 | galectin 3 binding protein |
| hemoglobin subunit delta | immunoglobulin kappa constant | keratin 2 | lectin, mannose binding 2 |
| hemoglobin subunit gamma 1 | immunoglobulin kappa variable 1-5 | keratin 3 | lipoprotein(a) |
| hexosaminidase subunit alpha | immunoglobulin kappa variable 1-12 | keratin 5 | lipoprotein(a) like 2, pseudogene |
| hexosaminidase subunit beta | immunoglobulin kappa variable 1-27 | keratin 9 | LDL receptor related protein 1 |
| HGF activator | immunoglobulin kappa variable 2-24 | keratin 10 | limbic system associated membrane protein |
| haptoglobin | immunoglobulin kappa variable 2-40 | keratin 13 | latent transforming growth factor beta binding protein 4 |
| hemopexin | immunoglobulin kappa variable 3-15 | keratin 14 | lumican |
| histidine rich glycoprotein | immunoglobulin kappa variable 3-20 | keratin 16 | lysozyme |
| hornerin | immunoglobulin kappa variable 3D-11 | keratin 17 | mannosidase alpha class 1A member 1 |
| heat shock protein 90 alpha family class A member 1 | immunoglobulin kappa variable 3D-20 | keratin 31 | mannosidase alpha class 2A member 2 |

## Slide 7
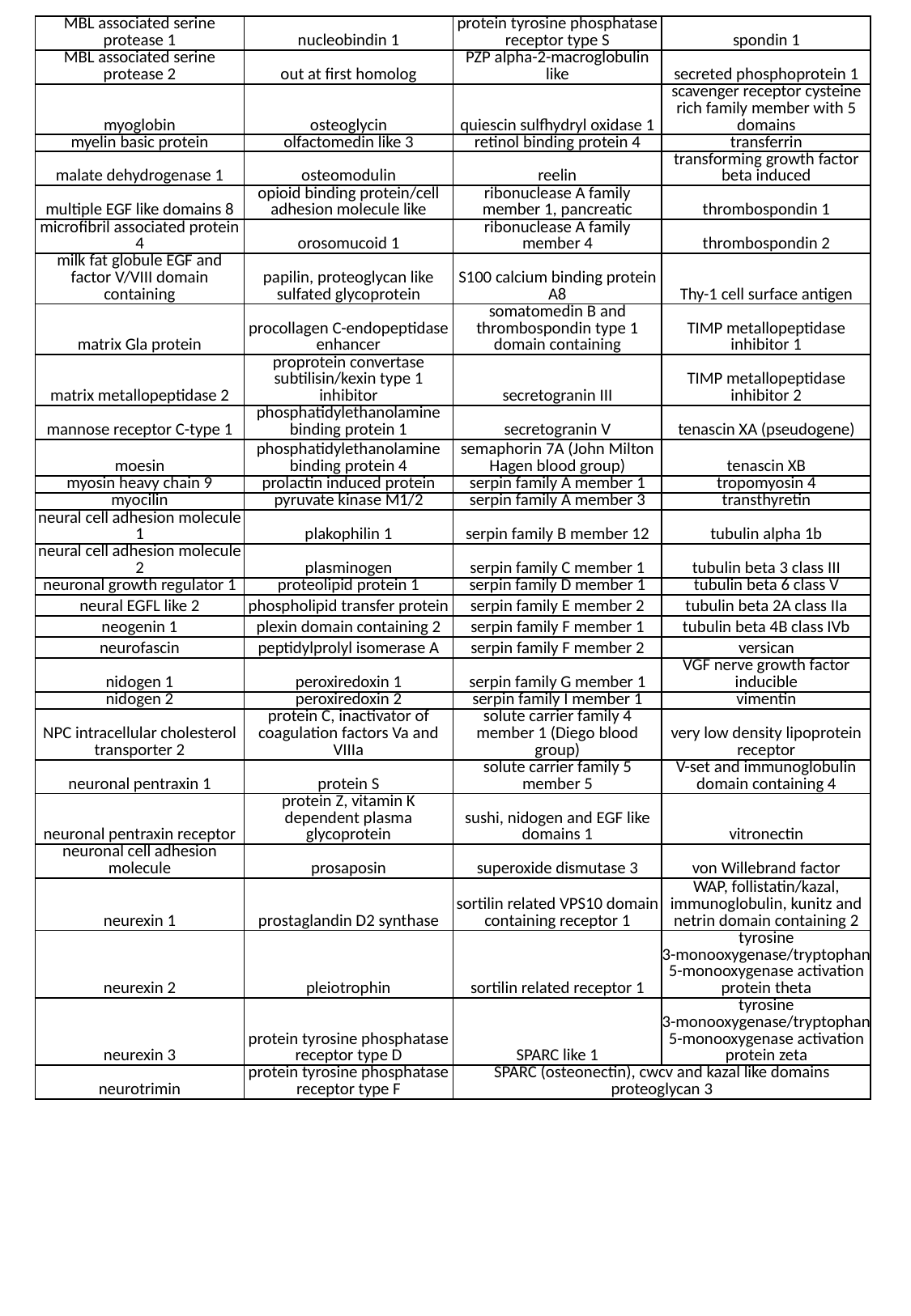

| MBL associated serine protease 1 | nucleobindin 1 | protein tyrosine phosphatase receptor type S | spondin 1 |
| --- | --- | --- | --- |
| MBL associated serine protease 2 | out at first homolog | PZP alpha-2-macroglobulin like | secreted phosphoprotein 1 |
| myoglobin | osteoglycin | quiescin sulfhydryl oxidase 1 | scavenger receptor cysteine rich family member with 5 domains |
| myelin basic protein | olfactomedin like 3 | retinol binding protein 4 | transferrin |
| malate dehydrogenase 1 | osteomodulin | reelin | transforming growth factor beta induced |
| multiple EGF like domains 8 | opioid binding protein/cell adhesion molecule like | ribonuclease A family member 1, pancreatic | thrombospondin 1 |
| microfibril associated protein 4 | orosomucoid 1 | ribonuclease A family member 4 | thrombospondin 2 |
| milk fat globule EGF and factor V/VIII domain containing | papilin, proteoglycan like sulfated glycoprotein | S100 calcium binding protein A8 | Thy-1 cell surface antigen |
| matrix Gla protein | procollagen C-endopeptidase enhancer | somatomedin B and thrombospondin type 1 domain containing | TIMP metallopeptidase inhibitor 1 |
| matrix metallopeptidase 2 | proprotein convertase subtilisin/kexin type 1 inhibitor | secretogranin III | TIMP metallopeptidase inhibitor 2 |
| mannose receptor C-type 1 | phosphatidylethanolamine binding protein 1 | secretogranin V | tenascin XA (pseudogene) |
| moesin | phosphatidylethanolamine binding protein 4 | semaphorin 7A (John Milton Hagen blood group) | tenascin XB |
| myosin heavy chain 9 | prolactin induced protein | serpin family A member 1 | tropomyosin 4 |
| myocilin | pyruvate kinase M1/2 | serpin family A member 3 | transthyretin |
| neural cell adhesion molecule 1 | plakophilin 1 | serpin family B member 12 | tubulin alpha 1b |
| neural cell adhesion molecule 2 | plasminogen | serpin family C member 1 | tubulin beta 3 class III |
| neuronal growth regulator 1 | proteolipid protein 1 | serpin family D member 1 | tubulin beta 6 class V |
| neural EGFL like 2 | phospholipid transfer protein | serpin family E member 2 | tubulin beta 2A class IIa |
| neogenin 1 | plexin domain containing 2 | serpin family F member 1 | tubulin beta 4B class IVb |
| neurofascin | peptidylprolyl isomerase A | serpin family F member 2 | versican |
| nidogen 1 | peroxiredoxin 1 | serpin family G member 1 | VGF nerve growth factor inducible |
| nidogen 2 | peroxiredoxin 2 | serpin family I member 1 | vimentin |
| NPC intracellular cholesterol transporter 2 | protein C, inactivator of coagulation factors Va and VIIIa | solute carrier family 4 member 1 (Diego blood group) | very low density lipoprotein receptor |
| neuronal pentraxin 1 | protein S | solute carrier family 5 member 5 | V-set and immunoglobulin domain containing 4 |
| neuronal pentraxin receptor | protein Z, vitamin K dependent plasma glycoprotein | sushi, nidogen and EGF like domains 1 | vitronectin |
| neuronal cell adhesion molecule | prosaposin | superoxide dismutase 3 | von Willebrand factor |
| neurexin 1 | prostaglandin D2 synthase | sortilin related VPS10 domain containing receptor 1 | WAP, follistatin/kazal, immunoglobulin, kunitz and netrin domain containing 2 |
| neurexin 2 | pleiotrophin | sortilin related receptor 1 | tyrosine 3-monooxygenase/tryptophan 5-monooxygenase activation protein theta |
| neurexin 3 | protein tyrosine phosphatase receptor type D | SPARC like 1 | tyrosine 3-monooxygenase/tryptophan 5-monooxygenase activation protein zeta |
| neurotrimin | protein tyrosine phosphatase receptor type F | SPARC (osteonectin), cwcv and kazal like domains proteoglycan 3 | |
